# Molecular analysis of infant fecal microbiota in an Asian at-risk cohort–correlates with infant and childhood eczema

**DOI:** 10.1186/1756-0500-7-166

**Published:** 2014-03-20

**Authors:** Gaik Chin Yap, Evelyn Xiu Ling Loo, Marion Aw, Qingshu Lu, Lynette Pei-Chi Shek, Bee Wah Lee

**Affiliations:** 1 Department of Paediatrics, Yong Loo Lin School of Medicine, National University of Singapore, Singapore, Singapore; 2 Singapore Clinical Research Institute, Singapore, Singapore

## Abstract

**Background:**

Studies have suggested that selective microbial targets prevail in the fecal microbiota of infants with eczema. This study evaluated the composition of fecal microbiota of infants who developed eczema in the first 5 years of life and compared these with those of healthy controls.

**Findings:**

Children who developed eczema in the first 2 years, those with eczema at 5 years of age and healthy controls were selected from the placebo arm of a birth cohort of at-risk infants participating in a randomized double-blind trial on the protective effects of supplemental probiotics in early life on allergic outcomes. Molecular evaluation of fecal microbiota were conducted using Fluorescence In Situ Hybridization-Flow Cytometry (FISH-FC) for fecal samples collected. Longitudinal analysis of fecal microbiota composition at three days, one and three months and one year of life revealed higher abundance of Enterobacteriaceae [coefficient (B): 1.081, 95% CI: 0.229-1.933, adj p = 0.014] and *Clostridium perfringens* [coefficient (B): 0.521, 95% CI: 0.556-0.988, adj p = 0.03] in those who developed eczema in the first 2 years life. In those with eczema at 5 years of age, a lower abundance of *Bifidobacterium* was observed [coefficient (B): -27.635, 95% CI: -50.040 - -5.231, adj p = 0.018].

**Conclusions:**

The differences in infant fecal microbiota observed in eczema subjects in this study support the notion that relative abundance of selective microbial targets may contribute to the subsequent development of eczema in childhood.

## Findings

### Background

Allergic diseases, such as eczema, are chronic inflammatory disorders with increasing global trends [1]. Intestinal microbiota play a role in the regulation of innate and adaptive immunity [2] and has been implicated in the development of allergy diseases [3]. The composition of fecal microbiota of infants and young children with eczema differ from healthy children [4]. This study aims to evaluate and monitor the composition, maturation, development of fecal microbiota at 4 times points of an at risk birth cohort in the first year of life, and compare these findings in relation to the development of eczema at 2 and 5 years of age with those of healthy controls.

### Methods

**Figure 1:**
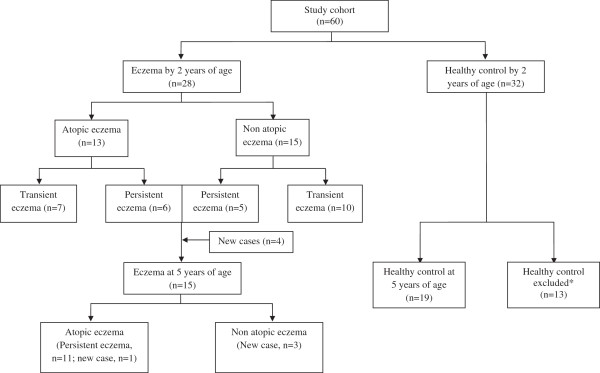
**Flowchart of study subjects showing the progress of eczema development in the first 2 years and at 5 years of age.** *Thirteen controls were excluded in the 5 year analysis because they developed allergen sensitization, rhinitis and wheeze at 5 years of age.

Subjects with eczema and healthy controls were selected from the placebo arm of a birth cohort of at-risk infants participating in a randomized double-blind trial on the protective effects of supplemental probiotics in early life on allergic outcomes [5]. Subjects who developed eczema in the first 2 years (eczema, n = 28; healthy, n = 32), and those with eczema at 5 years of age (eczema, n = 15 [persistent from 2 years: n = 11; new cases: n = 4]; healthy, n = 19) were studied. Thirteen controls were excluded in the 5 year analysis because they developed allergen sensitization, rhinitis and wheeze at 5 years of age (Figure 1). Informed consent was obtained from all families. The study was approved by the hospital’s institutional ethical review board (Ref Code: 2006/00008). Stool samples were collected at 4 time points (3 days, 1, 3 and 12 months of age) as previously described [6] There were missing stool samples in 2% to 25% of subjects at different time points, but these were not different between cases and controls. Subjects with eczema in the first 2 years and at 5 years of age were subclassified into atopic (positive skin prick test to common allergens) eczema (2 yrs: n = 13; 5 yrs: n = 12) and non-atopic eczema (2 yrs: n = 15; 5 yrs: n = 3).

Molecular evaluation of fecal microbiota was conducted using/harnessing whole-cell-based detection approach based on Fluorescence In Situ Hybridization combined with Flow Cytometry (FISH-FC) for fecal samples collected at 3 days, 1, 3 and 12 months. 16S rRNA probes were selected to target and quantify the *Eubacterium rectale-Clostridium coccoides* group (Erec 482), *Clostridium leptum* subgroup (Clep 866 and the corresponding competitor probes), *Bacteroides-Prevotella* group (Bac 303), *Bifidobacterium* genus (Bif 164), *Atopobium* group (Ato 291), *Lactobacilli-Enterococci* group (Lab 158), Enterobacteriaceae family (Enter 1432), *Clostridium perfringens* (Cperf191), *Clostridium difficile* (Cdif198) and *Escherichia coli* (Eco 1531) as previously described [7].

Linear mixed model was used to evaluate the longitudinal differences (i.e. 4 time points) of bacterial targets with adjustments for gender, mode of delivery, breastfeeding up to 6 months and comorbidities (rhinitis and wheeze) [8].

### Results

**Figure 2:**
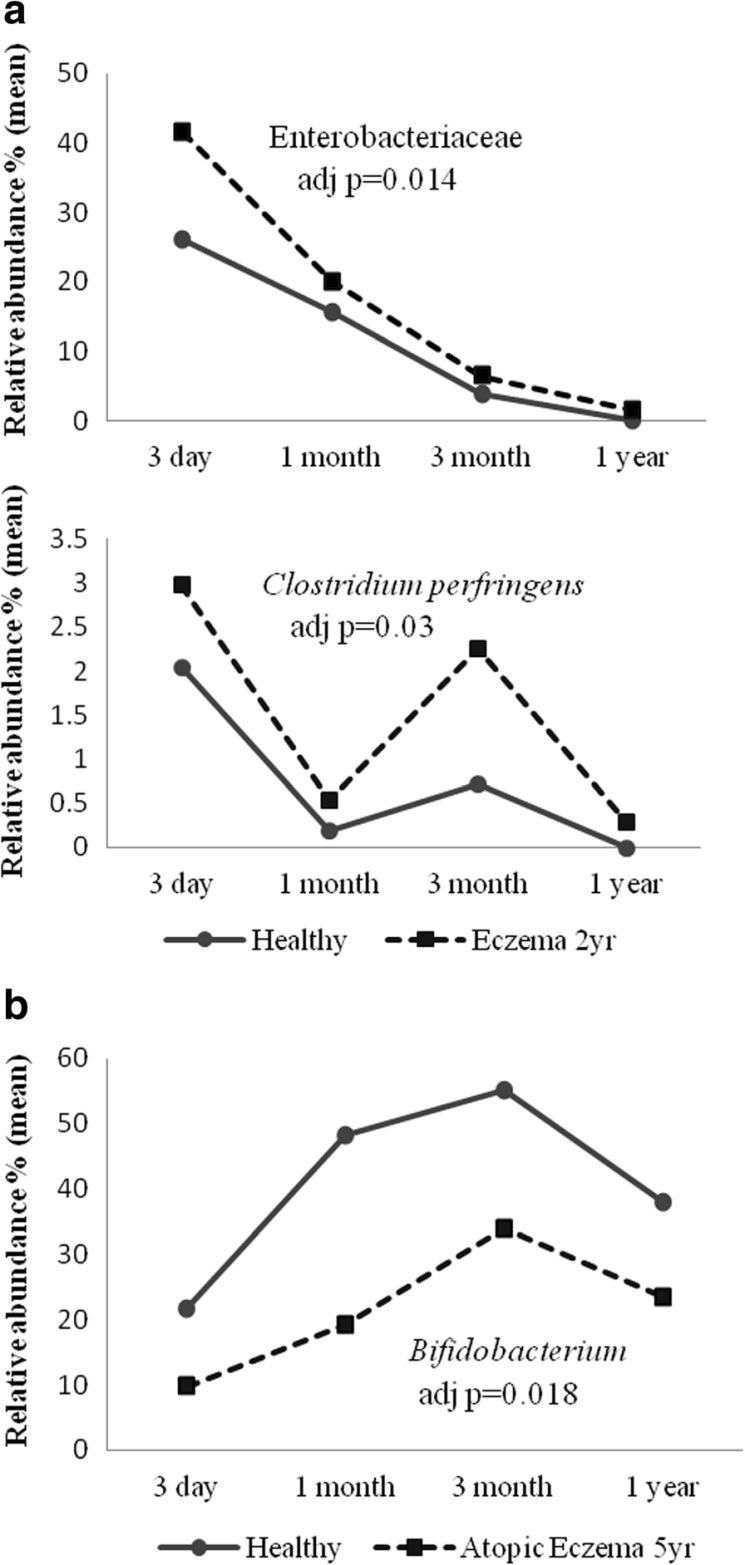
**Longitudinal comparison of the fecal microbiota of children with and without eczema.** Linear mixed model analysis of fecal microbiota of children with eczema in **(a)** the first 2 years of life and **(b)** at 5 years of age, compared to healthy controls. Only microbial targets with statistically significant difference between eczema and controls are shown.

The demographic characteristics and data showing the relative abundance of fecal bacterial groups for children who developed eczema by 2 and at 5 years of age and their healthy controls are summarized in Tables 1, 2 and 3 respectively. Longitudinal monitoring of the dynamics of intestinal bacterial colonization over 4 time points (3 days, one month, three months and one year of life) in the first year of life showed higher relative abundance of Enterobacteriaceae [coefficient (B): 1.081, 95% CI: 0.229-1.933, adj p = 0.014] in children with eczema by 2 years of age, and *Clostridium perfringens* [coefficient (B): 0.521, 95% CI: 0.556-0.988, adj p = 0.03] in eczema n = 28) by 2 years of age compared to healthy controls (n = 32) (Table 2 and Figure 2a).

**Table 1:** Demographic characteristics of children with eczema by 2 years and at 5 years of age and their healthy controls

	**By 2 years of age**			**At 5 years of age**		
	**Eczema (n = 28)**	**Healthy control (n = 32)**	**p value**	**Eczema (n = 15)**	**Healthy control (n = 19)**	**p value**
**Gender (%)**						
Male	14 (50)	15 (46.9)	0.809	6 (40)	9 (47.4)	0.667
Female	14 (50)	17 (53.1)		9 (60)	10 (52.6)	
**Mode of delivery (%)**						
Lower segment caesarean section	7 (25)	9 (28.1)	0.785	4 (26.7)	4 (21.1)	1
Vaginal delivery	21 (75)	23 (71.9)		11 (73.3)	15 (78.9)	
**Feeding history from birth to month 6 (%)**						
Breastfeeding and formula feeding	21 (75)	26 (81.3)	0.558	11 (73.3)	16 (84.2)	0.672
Total formula feeding	7 (25)	6 (18.7)		4 (26.7)	3 (15.8)	

**Table 2:** Relative abundance of fecal bacterial groups for children with eczema by 2 years of age and their healthy controls

		**Healthy**		**Eczema by 2 years of age**	
**Time points**	**Bacterial group**	**n**	**Mean (SD)**	**n**	**Mean (SD)**
3 day	*E.rectale - C. coccoides*	21	0.27 (1.139)	19	ND
	*Clostridium leptum*	21	3.08 (9.155)	19	ND
	*Bacteriodes-Prevotella*	21	5.56 (11.654)	19	6.88 (19.386)
	*Bifidobacterium*	21	29.35 (36.872)	19	15.88 (30.175)
	*Atopobium*	21	0.44 (0.801)	19	0.86 (3.555)
	*Lactobacilli–Enterococci*	21	3.22 (8.631)	19	0.49 (1.370)
	Enterobacteriaceae	21	26.22 (35.55)	19	41.64 (37.819)
	*Clostridium perfringens*	12	2.05 (7.093)	16	2.99 (8.137)
	*Clostridium difficile*	12	ND	16	0.66 (2.365)
	*E.coli*	11	14.17 (29.862)	16	34.98 (40.363)
1 month	*E.rectale–C. coccoides*	23	0.29 (0.589)	19	6.65 (18.510)
	*Clostridium leptum*	23	ND	19	2.47 (9.339)
	*Bacteriodes-Prevotella*	23	1.65 (3.309)	19	6.01 (16.408)
	*Bifidobacterium*	23	41.27 (38.514)	19	28.38 (33.945)
	*Atopobium*	23	6.52 (12.635)	19	2.32 (6.41)
	*Lactobacilli–Enterococci*	23	2.74 (7.265)	19	7.98 (15.762)
	Enterobacteriaceae	23	15.63 (23.726)	19	20.1 (23.837)
	*Clostridium perfringens*	15	0.19 (0.751)	18	0.55 (1.482)
	*Clostridium difficile*	15	ND	18	ND
	*E.coli*	15	9.01 (18.952)	18	3.62 (4.631)
3 month	*E.rectale–C. coccoides*	28	5.45 (10.551)	24	7.43 (13.389)
	*Clostridium leptum*	28	0.46 (1.590)	24	0.01 (0.059)
	*Bacteriodes-Prevotella*	28	3.53 (7.845)	24	3.32 (5.126)
	*Bifidobacterium*	28	55.86 (32.344)	24	54.21 (33.827)
	*Atopobium*	28	10.19 (16.06)	24	3.72 (7.037)
	*Lactobacilli–Enterococci*	28	2.77 (3.121)	24	2.62 (3.404)
	Enterobacteriaceae	28	3.8 (7.222)	24	6.51 (10.024)
	*Clostridium perfringens*	17	0.72 (1.547)	20	2.26 (6.246)
	*Clostridium difficile*	17	ND	20	ND
	*E.coli*	17	1.16 (2.725)	20	4.26 (10.987)
1 year	*E.rectale–C. coccoides*	27	30.40 (18.445)	24	31.25 (16.0.42)
	*Clostridium leptum*	27	4.22 (7.022)	24	1.33 (3.047)
	*Bacteriodes–Prevotella*	27	10.05 (10.082)	24	4.175 (6.049)
	*Bifidobacterium*	27	28.88 (23.227)	24	27.92 (25.196)
	*Atopobium*	27	5.91 (8.639)	24	5.05 (9.337)
	*Lactobacilli–Enterococci*	27	1.57 (3.437)	24	1.45 (4.99)
	Enterobacteriaceae	27	0.10 (0.3)	24	1.65 (3.162)
	*Clostridium perfringens*	18	ND	24	0.30 (1.457)
	*Clostridium difficile*	18	ND	22	0.07 (0.328)
	*E.coli*	18	0.05 (0.129)	23	0.83 (2.22)

ND, not detected.

**Table 3:** Relative abundance of fecal bacterial groups for children with eczema at 5 years of age and their healthy controls

		**Healthy**		**Eczema at 5 years of age**	
**Time points**	**Bacterial group**	**n**	**Mean (SD)**	**n**	**Mean (SD)**
3 day	*E.rectale–C. coccoides*	10	ND	9	ND
	*Clostridium leptum*	10	2.7 (8.526)	9	3.74 (11.217)
	*Bacteriodes-Prevotella*	10	10.32 (15.729)	9	7.49 (19.590)
	*Bifidobacterium*	10	21.65 (33.2)	9	24.45 (34.194)
	*Atopobium*	10	0.21 (0.439)	9	0.12 (0.229)
	*Lactobacilli–Enterococci*	10	5.48 (12.272)	9	0.64 (1.307)
	Enterobacteriaceae	10	47.73 (41.03)	9	30.89 (39.958)
	*Clostridium perfringens*	7	ND	5	ND
	*Clostridium difficile*	7	ND	5	ND
	*E.coli*	7	21.87 (35.99)	5	20.36 (39.873)
1 month	*E.rectale–C. coccoides*	13	0.32 (0.670)	10	0.74 (1.687)
	*Clostridium leptum*	13	ND	10	4.06 (12.829)
	*Bacteriodes-Prevotella*	13	2.92 (4.008)	10	6.56 (20.824)
	*Bifidobacterium*	13	48.29 (36.270)	10	21.28 (28.332)
	*Atopobium*	13	5.15 (9.403)	10	6.01 (12.463)
	*Lactobacilli–Enterococci*	13	1.91 (3.987)	10	5.77 (18.246)
	Enterobacteriaceae	13	19.54 (28.826)	10	18.19 (20.289)
	*Clostridium perfringens*	10	0.29 (0.92)	6	ND
	*Clostridium difficile*	10	ND	6	ND
	*E.coli*	10	11.62 (22.528)	6	5.27 (7.354)
3 month	*E.rectale–C. coccoides*	16	4.83 (10.187)	12	7.67 (14.804)
	*Clostridium leptum*	16	0.17 (0.688)	12	0.2 (0.678)
	*Bacteriodes-Prevotella*	16	2.83 (5.904)	12	6.42 (9.913)
	*Bifidobacterium*	16	55.21 (33.0)	12	42.557 (35.960)
	*Atopobium*	16	12.73 (19.887)	12	5.96 (9.282)
	*Lactobacilli–Enterococci*	16	2.79 (3.085)	12	3.07 (3.726)
	Enterobacteriaceae	16	4.28 (6.634)	12	10.94 (14.169)
	*Clostridium perfringens*	12	0.98 (1.791)	7	6.50 (9.55)
	*Clostridium difficile*	12	ND	7	ND
	*E.coli*	12	1.64 (3.152)	7	6.83 (13.998)
1 year	*E.rectale–C. coccoides*	15	28.20 (19.361)	12	29.78 (15.534)
	*Clostridium leptum*	15	3.54 (7.102)	12	1.84 (2.359)
	*Bacteriodes-Prevotella*	15	9.22 (10.450)	12	4.86 (8.232)
	*Bifidobacterium*	15	37.97 (23.939)	12	18.88 (24.0)
	*Atopobium*	15	6.45 (9.575)	12	4.40 (5.199)
	*Lactobacilli–Enterococci*	15	1.59 (3.724)	12	3.29 (6.987)
	Enterobacteriaceae	15	0.04 (0.152)	12	1.59 (3.133)
	*Clostridium perfringens*	12	ND	9	ND
	*Clostridium difficile*	12	ND	7	0.22 (0.582)
	*E.coli*	12	0.07 (0.155)	8	1.78 (3.521)

ND, not detected.

Sub-group analysis of atopic eczema (n = 13) and non-atopic eczema (n = 15) cases with onset in first 2 years of life yielded similar results, with higher abundance of Enterobacteriaceae compared to controls [atopic eczema: coefficient (B): 0.949, 95% CI: 0.214-1.683, adj p = 0.013; non atopic eczema: coefficient (B): 1.119, 95% CI: 0.136-2.102, adj p = 0.027], and *Clostridium perfringens* for non-atopic eczema only [coefficient (B): 0.713, 95% CI: 0.124-1.303, adj p = 0.022]. This analysis was also carried out with controls (n = 19) who were healthy all the way till 5 years of age (that is did not develop eczema after the age of 2 years), and similar findings were observed (data not shown).

For atopic eczema at 5 years old, a lower relative abundance of *Bifidobacterium* was found in cases (n = 15) compared to healthy controls (n = 19) [coefficient (B): -27.635, 95% CI: -50.040 - -5.231, adj p = 0.018] (Table 3 and Figure 2b).

### Discussion

In this study significant differences in fecal microbiota signatures analyzed at 4 times points in the first year of life were observed in infants who developed eczema in the first 5 years of life. These data suggests that specific microbial signatures appearing in early life might be predictive of eczema. Previous studies have also compared infant microbiota at more than one time point in infancy [3,9]. Sjögren et al. reported lower prevalence of lactobacilli group I and *Bifidobacterium adolescentis* at 1 week after birth for atopic dermatitis compared to non-allergic controls, but no significant differences were found for the other time points i.e. 1 month and 2 months [3]. In another study, reduced microbial diversity at 1 month and 12 months were associated with the presence of serum specific IgE, while allergic rhinitis group at school age showed less microbial diversity at 1 month [9]. Instead of making comparisons between single time points, our study utilized linear mixed model analysis to enabled comparisons over 4 time points in the first year of life. Our findings indicate that a consistent pattern in profile of fecal microbiota over the first year was associated with development of childhood eczema.

Our findings support the single time point cross sectional studies, where increased numbers of bacterium from *Clostridium* cluster IV and XIVa have been reported. These clusters of Clostridium are normally found in abundance in the adult intestine [10,11]. Although we did not find any difference for *Clostridium* cluster IV and XIVa in our study but we did observe an increase in *Clostridium perfringens* in children with eczema. This findings was supported by a recent study where Clostridium cluster I was associated with higher risk of developing atopic dermatitis [12].

An increased abundance of Enterobacteriaceae has also been observed in atopic subjects [13]. We have also previously shown by 16S rRNA pyrosequencing in this same cohort that fecal Enterobacteriaceae was more abundant at the age of 1 month in eczema compared to match controls [14]. Higher abundance of Enterobacteriaceae has been reported to be associated with intestinal inflammation (dextran sodium sulfate induced colitis), suggesting that Enterobacteriaceae may be responsible for promoting immune dysregulation [15].

The reduced abundance of *Bifidobacterium* in eczema compared to healthy controls has been observed in several other studies [3,7], and is associated with an increased risk of atopic dermatitis [16]. We did not observe reduced abundance of *Bifidobacterium* for eczema in the first 2 years of life compared to healthy controls. However, in subjects whose onset of eczema occurred by the age of 2 years (n = 28), we found that in those with eczema persistent till 5 years (n = 11) had lower abundances of *Bifidobacterium* compared with those with transient eczema (resolved before 5 years) (n = 17) as well as healthy controls, but these differences did reach statistical significance (p = 0.099) (data not shown). These observed differences, occurring in early life, in the maturation and development of the infant gut microbiota between subjects with atopic dermatitis and healthy controls suggests that the gut microbiota may influence immune system development [17] and contribute to disease severity [2].

In conclusion, this prospective study which profiled the dynamics of intestinal bacterial colonization over infancy supports the notion that relative abundance of selective microbial targets may contribute to the subsequent development of eczema in childhood.

## Competing interests

The authors declare that they have no competing interests.

## Authors’ contributions

GCY performed the experiments, data analysis and statistical analysis. EXLL drafted the manuscript. GCY and BWL helped to revise the manuscript. QSL participated in collation of clinical data. MA, LPCS and BWL participated in the study design and helped in coordination of sample and clinical data collection. All authors read and approved the final manuscript.
